# Fabrication and testing of a multifunctional SiO_2_@ZnO core–shell nanospheres incorporated polymer coating for sustainable marine transport

**DOI:** 10.1038/s41598-023-39423-9

**Published:** 2023-07-29

**Authors:** Jaya Verma, Yanquan Geng, Jiqiang Wang, Saurav Goel

**Affiliations:** 1 School of Engineering, London South Bank University, London, SE1 0 AA UK; 2 Center for Precision Engineering, Harbin Institute of Technology, Harbin, 150001 Heilongjiang China; 3 Department of Mechanical Engineering, University of Petroleum and Energy Studies, Dehradun, 248007 India

**Keywords:** Mechanical engineering, Nanoscale materials

## Abstract

We report the development of a coating system relying on the incorporation of SiO_2_@ZnO core–shell nanospheres in polyurethane media as a novel approach to achieve longevity and sustainability in marine transport. This polymeric coating showed significant improvement in surface abrasion resistance, the transition from a hydrophilic state to a hydrophobic state (~ 125.2° ± 2°), improved antifungal, antibacterial and antialgae effects which make the proposed coating ideal to protect steel surfaces against biofouling. To substantiate our claims, we performed X-ray diffraction, Transmission electron microscopy, Fourier transform infrared spectroscopy, scanning acoustic microscopy, Thermogravimetric analysis (TGA), contact angle measurements, antimicrobial (antialgal, antibacterial, antifungal) tests and Taber abrasion tests (ASTM D1044 and D4060) to highlight the mechanical and biological functionality as well as the bonding configuration of this coating. The wear analysis of the Taber abraded coating using SEM and optical microscopy showed significant improvement in the adhesion and shear resistance achieved by the SiO_2_@ZnO core–shell nanospheres incorporated PU coating which was a contrasting feature compared to using PU alone. The overall investigations we performed led us to find out that the addition of 4% (wt.) SiO_2_@ZnO core–shell nanoparticles to the PU media deposited on the low carbon steel surface demonstrated remarkable antimicrobial performance with almost no bacterial growth, significant reductions in growth for algae to about 90% and fungus to about 95%.

## Introduction

Surfaces of various products used commercially such as automotive and commercial transport vehicles, offshore and onshore oil and gas structural steel, vessels, accommodation modules, pipeline externals, retail and commercial architectural and structural steelwork are covered with polymer coatings to provide them better functionality. Polymeric coatings can be protective (anticorrosive), aesthetic (paint), or add new functionality (adhesives, photographic films). Polymeric coatings, which are mostly made of organic materials, when mixed with metals or ceramics or a combination of them results in the formation of nanocomposite coatings which can further enhance the utility and durability of a component^1,2^. High-performance, super-durable coatings are needed for outdoor applications since photo-degradation reduces the endurance of a polymer coating. A new materials innovation that has started to appear in the arena of coating systems is the mixing of core–shell nanospheres/nanoparticles in a parent media. A core–shell nanosphere is essentially a composite nanoparticle having a solid or a hollow core in the inner and another material deposited as a shell. Thus, two states of matter are derived which are interspersed with a third state of matter such as a polymer media in a certain weight percentage to achieve an avalanche of new functionalities that cannot be obtained either by the parent matrix or by the core–shell materials alone^3^. The schematic diagram illustrating the fundamental concept of core–shell nanoparticles can be seen from the supplementary information (see Fig S1).

Core–shell (CS) is a biphase material with an inner core structure and an exterior shell. Thus, core/shell nanoparticles are functional materials that can be tuned to achieve desirable properties^4^. Sometimes, the characteristics resulting from the core or shell materials can be very dissimilar. Either the constituent materials or the core to shell ratio can be changed to alter the characteristics. The attributes of the core particle, such as reactivity or thermal stability, can be altered by the shell material coating, increasing the core particle's stability and dispersibility. The processed core–shell particles exhibit distinctive qualities. This is particularly true regarding the inherent capability to adjust the surface functionalities to satisfy the various application needs. The shell formation on the core particle serves a variety of purposes, including surface modification, the ability to improve functionality, stability, dispersibility, controlled core release, reduction in precious material consumption, and others^5^.

A wide range of materials to date are incorporated through a core–shell (CS) approach including SiO_2_, Al_2_O_3_, TiO_2_, ZnO, and ZnS. ZnO and TiO_2_ are two nanomaterials most used as UV blockers. The effects of nano-ZnO addition on the UV-resistant qualities of polyurethane/acrylic coatings have been the topic of recent investigations^6,7^. To date, several polymers have been considered to develop the CS incorporated coatings with each having their unique benefits. This work extends the extant understanding of the topic by incorporating SiO_2_@ZnO CS into the polyurethane (PU) polymer media used as a nanocomposite coating on top of the steel substrates to achieve sustainability in marine transportation^8^. The polymeric coatings often contain polyurethane (PU) resins, which are typically created through the reaction of polyalcohol and an organic di-isocyanate^9^. Coatings made through this pathway become useful for functional and mechanical protection for products in paints, food, energy saving, glazing and corrosion protection. This occurs by changed contact angle/wetting which in turn leads to desirable hydrophilic, hydrophobic, or superhydrophobic characteristics^10^. Through the addition of metal oxide nanocomposites, these PU-based coatings can improve their efficacy for multipurpose applications^11^.

The current work was driven by the need to harness the synergistic effects of achieving high mechanical strength of SiO_2_ (core)^12^ and antimicrobial characteristics of ZnO (shell)^13^. It was anticipated that the mixing of CS in the PU polymer will create a level of surface protection that will surmount the reported benefits derived from the SiO_2_@TiO_2_ CS + PU coating^1,14–16^, the best-in-class coating reported to date. The amorphous silicon dioxide (SiO_2_) has a very unique structure that manifests odd or aberrant physical and chemical properties^17,18^. Due to its numerous fantastic features, including high strength, high toughness and exceptional stability, SiO_2_ nanoparticles can be used widely for a lot of engineering applications. ZnO as a shell on top of the SiO_2_ core is an important addition because of its numerous desirable properties, including its high catalytic activity, physical and chemical stability and antimicrobial capabilities^19,20^. The low price and high photochemical reactivity of ZnO are complemented by its broad band gap of 3.37 eV and large free exciton binding energy of 60 meV^21^. ZnO has been used in medical applications as a highly efficient antimicrobial chemical agent, drug transporter and bio-imaging probes since it is biosafe and biocompatible^22,23^. It was therefore deemed necessary to test the hypothesis of design and fabrication of SiO_2_@ZnO core–shell nanospheres to be incorporated into the PU media for the creation of abrasion-resistant, antibacterial, antifungal, antialgal and hydrophobic coating that can be used in marine transport. It was anticipated that the incorporation of SiO_2_@ZnO nanospherical core-shells improve the performance of the polymeric coating in three ways: (i) core silica provides mechanical strength due to its high mechanical stiffness; (ii) shell ZnO improves wetting properties that help to gain hydrophobicity and antimicrobial properties; and (iii) this layer becomes a barrier to prevent oxidation of the steel substrate which can slow the corrosion induced damage. Once synthesized, the coating can directly be applied with a paint brush which makes it an easily deployable solution to protect steel surfaces used in marine or saline environments.

## Experimental details

### Materials

The major consumables for the synthesis of core–shell SiO_2_@ZnO were Tetraethyl orthosilicate (TEOS), zinc acetate, triethanolamine, 3-aminopropyl triethoxysilane (APTES), polydimethylsiloxane (PDMS), ammonium hydroxide and xylene. These were procured from Sisco Research Laboratories Pvt. Ltd., India. Ethyl alcohol (C_2_H_5_OH) was procured from C.H. Fine Chemical Co., Ltd., Dibutyltin Dilaurate was purchased from TCI Chemicals Pvt. Ltd. and the Polyurethane media was purchased from Dalton Chemicals Pvt. Limited, India.

### Synthesis and fabrication of CS incorporated coating

#### Preparation of SiO_2_ nanoparticles

In this study, silica (SiO_2_) nanospheres were fabricated using the Stober process^24^, which involves the hydrolysis of tetraethyl orthosilicate in an ethanol solution with water and ammonia. In a typical experiment, 8 mL of tetraethyl orthosilicate was added to a mixture of 100 mL of ethanol and 35 mL of DI water. The solution was stirred for about 40 min. The resultant silica nanospheres were then ultrasonically cleaned with ethanol and centrifuged (at 8000 rpm) away from the suspension. Additionally, the material was dried at 100 °C for 24 h. and then calcined at 650 °C for two hours to produce nanopowder.

#### Synthesis of ZnO shell on SiO_2_ core

ZnO-coated SiO_2_ composites were produced by adding triethanolamine and Zinc Acetate (Zn(II)Ac2) to the SiO_2_ ethanol aqueous solution simultaneously. Typically, 0.2 g of SiO_2_ was dissolved in a 30 mL solution of ethanol and water (2:3) to prepare SiO_2_@ZnO. The SiO_2_ solution was then heated to 90 °C. After 10 min, a steady flow rate was maintained by the multichannel syringe pump as 1.6 mol/L triethanolamine and 0.02 mol/L Zn(II)Ac2 were simultaneously injected into the SiO_2_ ethanol aqueous solution through latex tubes. At 90 °C, the system was then continuously agitated for 1 h. The resulting white particles were centrifuged and rinsed repeatedly in doubly distilled water and were vacuum dried. The powders were then sintered for three hours at 700 °C.

#### Functionalisation of SiO_2_@ZnO nanospheres for thorough mixing in PU

**Figure 1:**
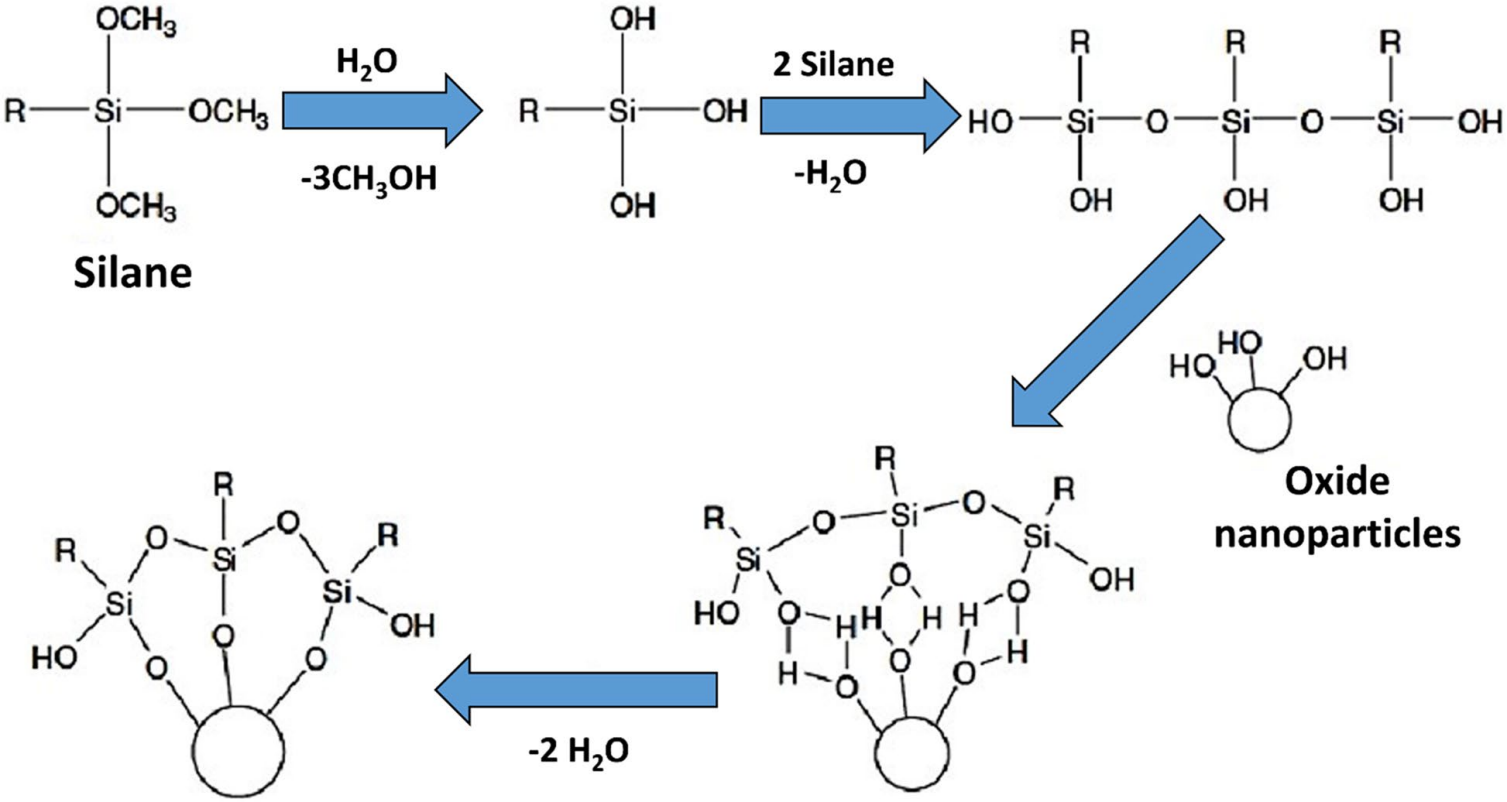
Protocol used for functionalization of nanoparticles.

To get neat bonding and thorough steering with core–shell nanostructures, surface functionalisation of the ZnO shell is crucial. This was accomplished as per the protocol shown in Fig. 1. The process of nano core–shell functionalisation involved dispersion of the material in xylene with a core/shell:xylene weight ratio of 1:4. This step created a slurry which was sonicated for 30 min to further improve the dispersion. PDMS and APTES were then added to the core/shell xylene suspension in a weight ratio of 3:1. The solution was then added with two drops of DBTDL catalyst while maintaining a temperature of 80 °C and stirring it at 1200 rpm. After obtaining the desired solution, it was sonicated for 40 min and dried overnight at 100 °C. A comparison of the two coatings seen through a camera is shown in Fig S2 as supplementary info to contrast the appearance of PU + SiO_2_@ZnO vs PU.

#### Coating formulation

Through probe sonication, PU binder, thinner and SiO_2_@ZnO core–shell nanoparticles were combined to create the SiO_2_@ZnO incorporated PU coatings. In the formulation, the binder to thinner ratio was set as 1:2. The coating formulation contained 1–4wt% nanoparticles in varying amounts which was one of the other objectives of the study i.e., to identify and screen the best performing loading percentage of SiO_2_@ZnO CS by optimization of its mix ratio. The coating was applied to the steel substrates (2.5 × 2 cm^2^) using a paintbrush with various concentrations (1–4wt%) and heated at 100 °C for 1 h. A completely dry coating was achieved with near uniform thickness of around 80 µm. The substrate used for applying the polymeric coating was a low carbon steel (Grade: 1006-1026^25^). A typical measurement obtained from the transmission electron microscope (TEM) showed that the silica core was about 90.3 nm diameter and the thickness of the ZnO shell on top of this core was about 32.7 nm. Thus, the total diameter of the CS of SiO_2_@ZnO prepared in this work was of the order of 155.7 nm.

#### Antimicrobial tests

##### Antialgal tests

In this study, we used green algae such as *Chlorella pyrenoidosa* and some mixed algae strains with *Oedogonium* sp. to assess the antialgal behaviour of the coating as a representative test for emulating sea water test conditions. These algae can have side effects on people as they can cause an allergic reaction such as rash, difficulty in breathing, swelling and anaphylaxis. Other potential side effects include goiter, skin reactions and gastrointestinal effects.

Isolation of algal strains: Algal strains were isolated and purified through repeated cultivation and culture on solidified and liquid media known as Fogg’s media as per the composition shown in Table 1.

**Table 1:** The composition of Fogg’s media.

Ingredient	mg/L
Potassium dihydrogen phosphate (K_2_HPO_4_)	0.2 g
Magnesium sulphate (MgSO_4_)	0.2 g
FE-EDTA stock solution	1.0 mL
Calcium chloride (CaCl_2_.2H_2_O)	0.1 g
Micronutrient solution	1.0 mL
KNO_3_	1.5 g
pH	6.5–7.0
Distilled water	1000 mL

About 1 mL of streptomycin was added to the liquid culture medium stored at 28 ± 2 °C in the culture room for two days to prevent the growth of bacterial contaminants. It was then used in little amounts and serially diluted. By using a glass rod and 1 mL of the diluted algal specimen, the nutrient agar plates’ surface was evenly covered. After that, the plates were incubated in the culture room until clearly defined colonies emerged. Colonies were selected, scooped up and then suspended in the brand-new liquid medium. Samples of 1 mL were once more plated on the nutrient agar plate and incubated in the culture room after 15 days of growth. Unique, healthy colonies were selected, and they were injected into an Erlenmeyer flask with fresh liquid media. The procedure was repeated until uni-algal culture was obtained.

Algal biomass culture and growth conditions: *Chlorella pyrenoidosa* (*C. pyrenoidosa*) and mixed algae (*C. pyrenoidosa* and *Oedogonium* sp.) were grown photoautotrophically in the sterile Fogg’s media with 5% algal biomass concentration. The cultures were raised in 1000 mL Erlenmeyer flasks with 500 mL media at 25 °C under fluorescent lighting (40 W of white light). Throughout the experiments, the culture concentration was held at 0.4 OD of algal biomass. For green (*C. pyrenoidosa*) and mixed algae (*C. pyrenoidosa* and *Oedogonium* sp.), the algal growth on coated samples was evaluated at 665 nm (A665nm) using a UV–vis spectrophotometer.

##### Antibacterial tests

In this study, antibacterial testing was performed on *Escherichia coli* (gram negative) and *Bacillus* (gram positive) bacteria which are representative marine environment bacteria.

Media preparation: The antibacterial tests were carried out by combining 7.5 g of nutritional broth with 500 mL of distilled water while stirring. This solution was autoclaved at 15 bars of pressure for 30 min at 121 °C to homogeneously sterilize it. The temperature was cooled to ambient conditions. 20 mL of this medium was divided among the test-tubes. Separate test tubes containing coated samples were introduced in the presence of *E. coli* and *Bacillus* bacteria. The coating nanoparticle composition ranged from 1% (wt) to 4% (wt).

##### Antifungal testing

In this study, antifungal activity was investigated using two types of pathogenic fungus namely, *acremonium* and *fusarium* species which are found mostly in soil, marine water and plant debris.

Media preparation: Antifungal testing was carried out by dissolving 39 g of PDA in 1000 mL of distilled water while stirring. This solution was autoclaved at 15 psi for 15 min at 121 °C to sterilise it. The media had nanoparticle concentrations ranging from 1% (wt) to 4% (wt). Fungus was observed to form on various petri-plates containing sterile media. The coated samples were placed in these Petri-plates, which were placed in a biochemical oxygen demand (BOD) chamber. Fungus growth was seen for up to 10 days.

## Results and discussions

The synthesized coated samples were thoroughly characterised for their microstructure, topography, chemical composition, mechanical abrasion resistance and various other functions using a wide range of characterization tools. These tools included examination using SEM, EDS, FTIR, TEM and XRD. Additionally, some functional tests were also performed to assess the coating using Taber Abrasion tests accompanied by optical microscopy to inspect the abraded debris of the coating, scanning acoustic microscopy for non-destructive evaluation of the microstructural state of the coating (qualitatively) and other functional tests such as measurement of contact angle, Thermal gravimetric analysis, antibacterial, antialgal and antifungal tests as per the standard experimental protocols.

### Scanning electron microscopy (SEM) and energy dispersive spectroscopy (EDS)

**Figure 2:**
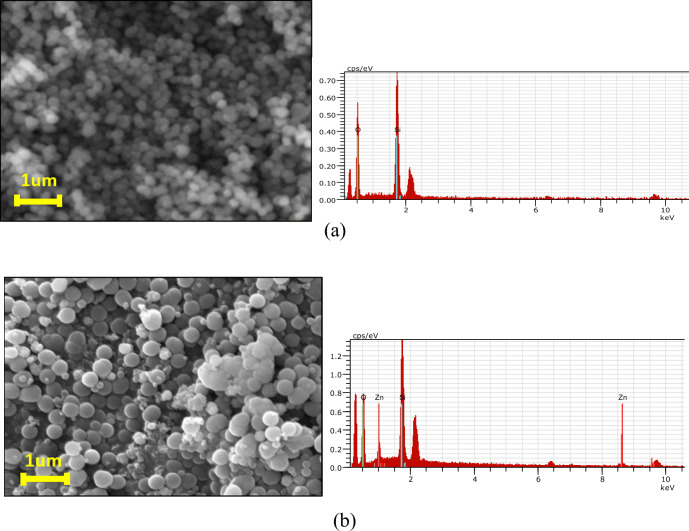
(**a**) SEM and EDS of SiO_2_ nanospheres. (**b**) SEM and EDS of SiO_2_@ZnO CS nanospheres synthesised in this work.

High-resolution SEM imaging was carried out by Zeiss SUPRA 55VP scanning electron microscopy. The SEM comes equipped with the energy dispersive X-ray analysis (EDS) which was used to assess the chemical details of the CS nanospheres. A typical SEM output of the SiO_2_ and SiO_2_@ZnO obtained in this work is compared in Fig. 2a, b. The SEM images highlight that most CS nanospheres fabricated in this work were spherical. Respective EDS inspection confirmed the presence of Zn, Si and O elements in the CS nanospheres. To further inspect the constituents of these nanospheres, FTIR analysis was done next.

### Fourier transform infrared spectroscopy (FTIR)

**Figure 3:**
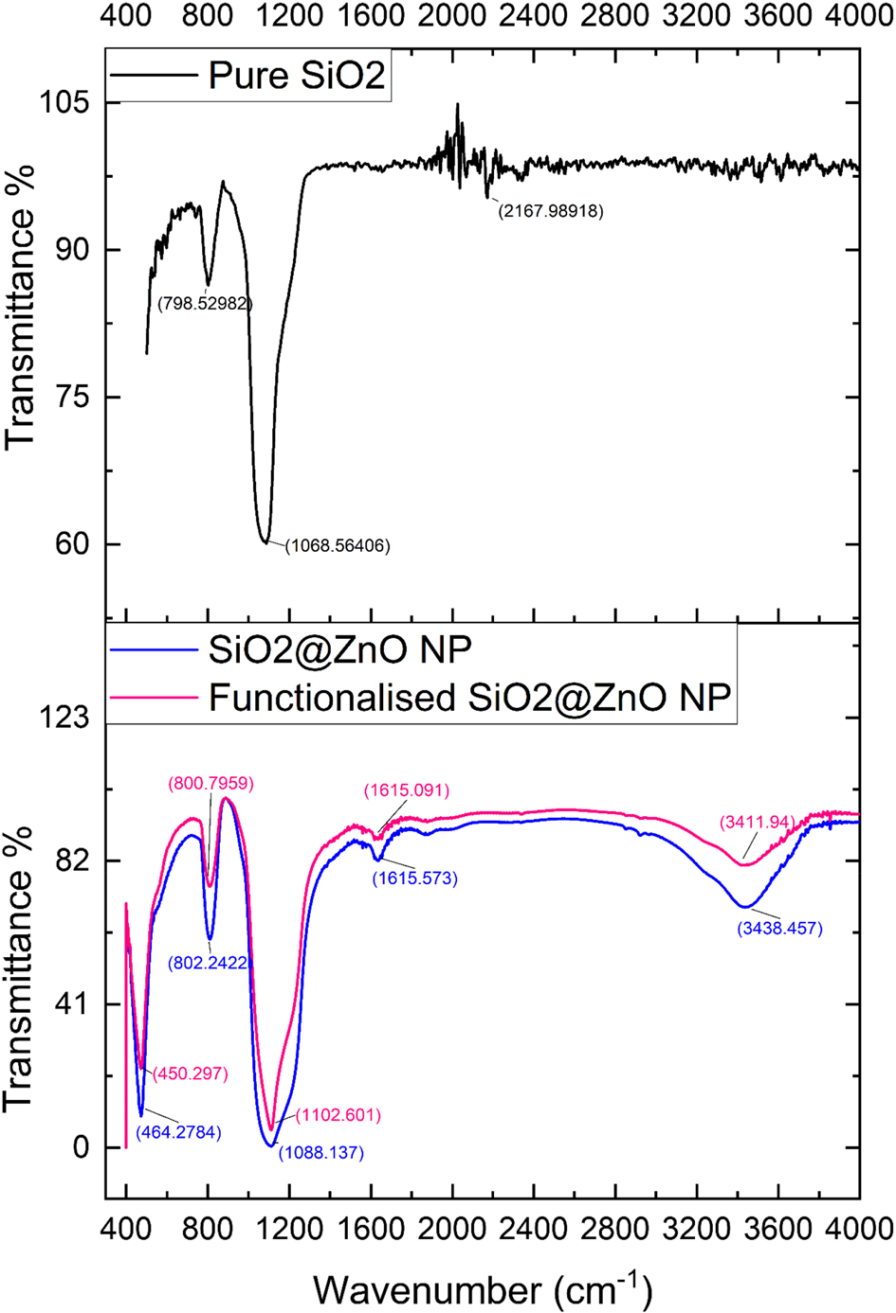
FTIR spectrum of core silica and silica@ZnO core–shell nanoparticles (functionalised and unfunctionalised). Image processed using Origin Software (https://www.originlab.com/).

FTIR spectrometer (Shimadzu 8400) was used to record the infrared absorption spectra of the SiO_2_ and CS nanospheres which are shown in Fig. 3. The development of an interfacial connection between SiO_2_ and ZnO can readily be seen from the infrared (IR) spectra of SiO_2_ and SiO_2_@ZnO. In the spectra of silica, the band around 1068 cm^−1^ corresponds to the asymmetric stretching vibration of the Si–O–Si bond whereas 2167 cm^−1^ bands appeared for H–O–H stretching and the symmetric stretching of the Si–O–Si group is represented by the band at 798 cm^−1^. The Si–O–Si bridge of the siloxane link’s asymmetric stretching vibrational mode corresponds to the broad doublet band found in the wavenumber range of 1300–1000 cm^−1^. The pronounced band at 1068 cm^−1^ is associated with the typical oxygen asymmetric stretching mode. Surface silanols and stretched siloxane linkages are likely to be arising due to the splitting of the asymmetric stretching mode (disorder-induced coupling). The asymmetric stretching mode of the SiO_2_ caused significant changes as seen in the IR spectra of SiO_2_@ZnO (Fig. 3). The sharp band near 1104 cm^−1^ corresponding to the asymmetric stretching mode showed a surface modification by the ZnO coating, replacing the doublet of the SiO_2_ asymmetric stretching band. After shell formation, the asymmetric stretching mode exhibits a 54 cm^-1^ shift to a higher frequency, which is likely due to the altered bonding around the [SiO4] tetrahedral structure. Once the ZnO gets coated on top of the SiO_2_ surface, a significant vibration ranging from 400 to 500 cm^−1^ was seen which can be assigned to the stretching characteristic stretching mode of the Zn–O bond. In the case of SiO_2_–ZnO core–shell nanoparticles, the intensity of the peak gets reduced due to the formation of a ZnO shell on the surface of SiO_2_ nanoparticles. A broad peak at 3438 cm^−1^ (stretching) and 1615 cm^−1^ (bending) indicated the presence of hydroxyl residue which is due to atmospheric moisture. By functionalising the surface of the core–shell with PDMS and APTES, the groups’ peak positions were almost similar and it’s just that the intensities of various peaks split due to the asymmetric stretching mode.

### X-ray diffraction analysis (XRD)

**Figure 4:**
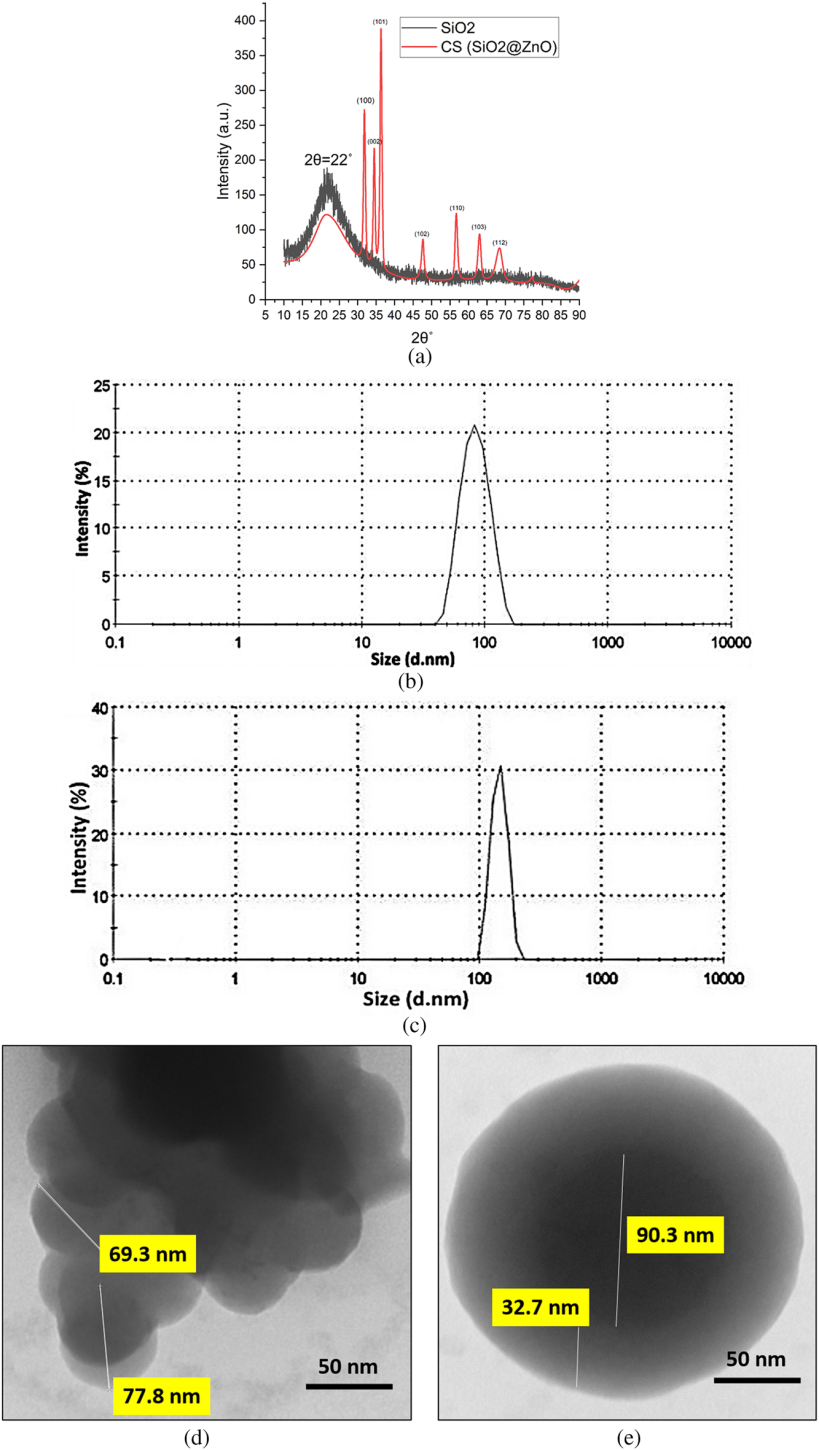
(**a**) XRD spectra for core SiO_2_ and SiO_2_@ZnO core–shell nanoparticle. (**b**) DLS of SiO_2_ (**c**) DLS of SiO_2_@ZnO core–shell nanoparticle (**d**) TEM image of the SiO_2_ (**e**) TEM image of SiO_2_@ZnO core–shell nanoparticle.

The X-ray diffraction was performed using Bruker D8 Advance to assess the chemical fingerprint of SiO_2_ and SiO_2_@ZnO CS nanospheres. The zinc-blende crystal structure of ZnO can be seen from the strong peaks shown in Fig. 4a which were resolved to obtain orientation information. The ZnO surface can be seen to possess a crystalline nature with sharp peaks identified at 31.8°, 34.5°, 36.3°, 47.72°, 56.8° and 63﻿° corresponding to the (100), (002), (101), (102), (110) and (103) orientations of ZnO. The SiO_2_ nanosphere showed just one broad peak at about 22° suggesting the material to have amorphous nature. The Debye-Scherer formula $D=\frac{K\uplambda }{\beta cos\theta }$ can be used to estimate the crystalline size (*D*) of the ZnO particle where *λ* is the wavelength, *θ* is the Bragg diffraction angle, *K* the Scherrer constant (0.98), and *β* is the peak width at half-maximum. From these formulae, the CS was estimated to have a size of 16.27 and 17.93 nm, respectively, according to the XRD peaks shown in Fig. 4a at ﻿36.3° and ﻿56.8°.

### Particle size distribution by dynamic light scattering

The particle size measurements were performed by Dynamic light scattering (DLS) zetasizer Nano S-90. The nanoparticles prepared were suspended in double distilled water by sonication. The light source used in the instrument was He–Ne laser light with a wavelength of 633 nm and a scattering angle of 90°. 1 mL of nanoparticle suspension was taken in a polystyrene cuvette and analyzed in the DLS instrument to determine particle size distribution and poly dispersity index. The temperature of the sample was maintained at 25 °C. Through DLS measurement, the SiO_2_ nanoparticle was estimated to be about 90 nm (dia) and the SiO_2_@ZnO core–shell nanoparticle was estimated to be around 170 nm (dia) (see Fig. 4b, c).

### Transmission electron microscopy (TEM)

The size of the SiO_2_@ZnO coreshell nanosphere was further confirmed through a direct examination using a 200 kV transmission electron microscope (TEM, Tecnai Osiris, FEI) which was equipped with a scanning unit (STEM) including high-angle annular dark-field (HAADF, Fischione Co.) detector and energy dispersive X-ray spectrometer (EDX, Super-X system with 4 Bruker silicon drift detectors, Thermo Fisher Co.).

Using a drop of sample suspension in doubly distilled water on a Formvar copper grid, samples were obtained for the TEM. The samples were then air dried to remove the solvent. The TEM image of SiO_2_ and SiO_2_/ZnO is shown in Fig. 4d, e. The individual core silica nanoparticles image shown in Fig. 4d indicated that the particle size was in the range of 70–77 nm. Figure 4e shows a closer view of the ZnO particle (light contrast) indicating that the ZnO shell surrounds the bare SiO_2_ core. The core showed a diameter of 90.3 nm with a shell thickness of 32.7 nm with no free bare zones. This analysis has a point resolution of 0.27 nm and was carried out at 20 kV and 100 times magnification.

### Physical attributes of SiO_2_@ZnO coating

#### Coating thickness measurements

**Figure 5:**
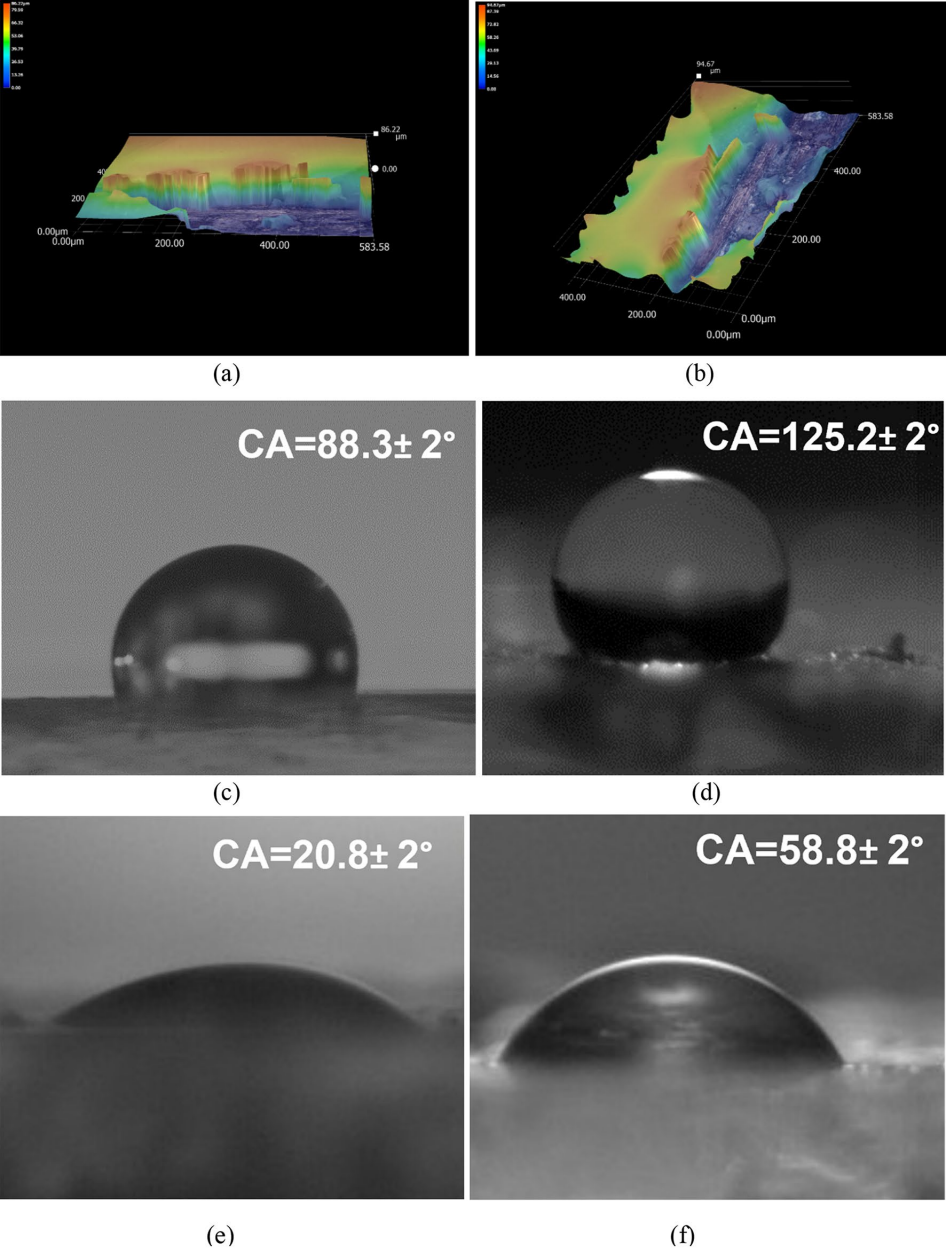
(**a**) and (**b**) showing coating thickness of ~ 80 µm. Contact angle measurement of coatings with water (**c**) PU alone (**d**) SiO_2_@ZnO incorporated PU core–shell coating. Contact angle measurement of coatings with oil (**e**) PU alone (**f**) SiO_2_@ZnO incorporated PU core–shell coating.

The thickness of the silica@ZnO core–shell incorporated PU coating was estimated using a digital microscope (Keyence VH-Z500R) following a scratch mark made on the coating by cleaving the polymer from the top of the steel. This destructive test revealed the coating thickness to be in the range of about ~ 80 µm and the results can be seen in Fig. 5a, b. Further tests on coatings such as surface wettability, antimicrobial activity, mechanical properties etc. are explained next.

#### Surface wettability and contact angle measurements

One of the important attributes of checking the change in the surface energy introduced by an agent mixed in the coating formulation is through the measurement of contact angle with different types of liquid such as water and oil^26–28^. We used DI water and rapeseed oil for surface wettability testing of the coatings developed in this work.

To perform this task, the sessile drop method with an OCA 25 goniometer was used (DataPhysics Instruments GmbH, Germany). The images of the contact angles were analysed with SCA20 software (DataPhysics Instruments GmbH, Germany). The usual method of performing this measurement entails dropping a controlled volume of DI water (droplet volume of 1 µL) on a given test surface. Three measurements were taken after 8–10 s of contact with the sample surface and the results are shown in Fig. 5c–f. The roughness corrected contact angle (Young’s contact angle) was calculated using $\mathrm{cos}{\theta }_{w}=r\mathrm{cos}{\theta }_{Y}$ and $r=1+\frac{{S}_{dr}}{100}$ where ${\theta }_{w}$ is the measured contact angle, ${\theta }_{Y}$ is Young’s contact angle, $r$ is the roughness factor, and ${S}_{dr}$ is the developed interfacial area ratio.

During the measurements, it was noticed that the addition of SiO_2_@ZnO CS nanospheres into the PU media enabled the transition from a hydrophilic state (10°  ≤ CA ≤ 90°) to a hydrophobic state (90° ≤ CA ≤ 150°) regardless of the wt% of CS (1–4% tested in this work) but this occurs only for the water media and not for the oil media. As for oil media, the improvement was insignificant and the contact angle was in the range of 10° ≤ CA ≤ 90°, which keeps both PU and PU + SiO_2_@ZnO as oleophilic to the oil. In particular, for the water contact angle, the best result was obtained for the 4% (wt) addition of core–shell nanoparticles to the PU media wherein the contact angle was about 105.4° ± 2° for the water drop before functionalisation, On functionalisation, it improved further to 125.2° ± 2° while pure PU coating showed a CA of about 88.3° ± 2° whereas the PU coating with just silica core showed a CA of about 86.4° ± 2° (Fig. 5c, d). These results indicated the effectiveness of the ZnO shell on core silica material in obtaining a hydrophobic surface. For oil, the contact angle was measured as 20.8° ± 2° for pure PU coating and 58.8° ± 2° with core–shell nanoparticles incorporated in PU after functionalisation (Fig. 5e, f). Thus, it can be concluded that the developed SiO_2_@ZnO incorporated PU coating showed hydrophobic behaviour for water but not for oil.

#### Thermal gravimetric analysis (TGA)

**Figure 6:**
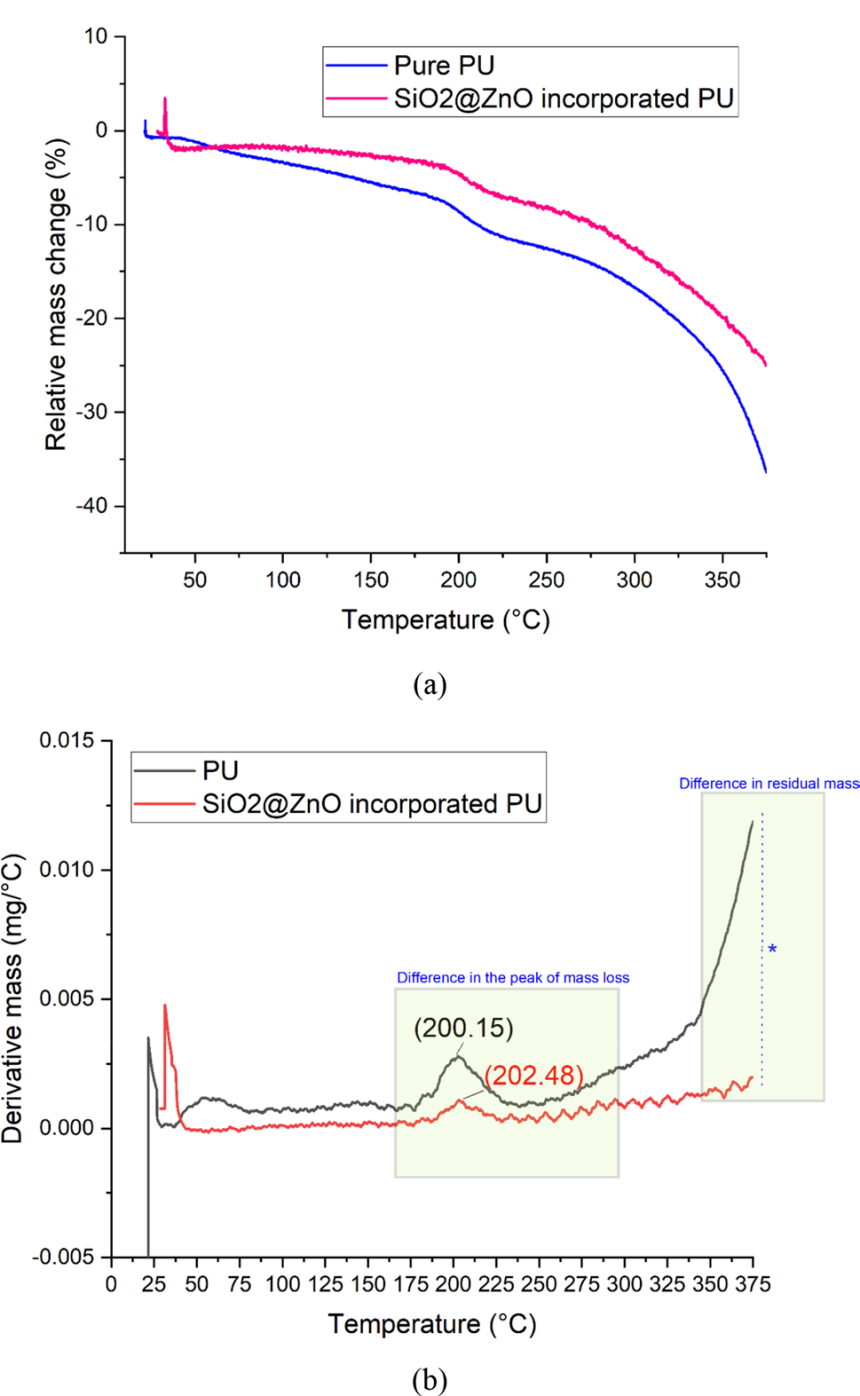
TGA analysis of PU and core–shell incorporated PU coatings showing change in (**a**) relative mass (%). (**b**) derivative mass (mg/°C). Image processed using Origin Software (https://www.originlab.com/).

The coatings' thermal gravimetric data is presented in Fig. 6a, b. The TGA thermograms demonstrate a sizable relative mass loss in percentage steps and derivative mass (mg/°C). The TGA data showed that the PU had the lowest T_onset_ value of 200.15 °C followed by PU/SiO_2_-ZnO of about 202.48 °C. The nanospheres present in the coating were the only main reason for the observed difference in the TGA of the two types of coatings. The measured onset degradation temperature increased with the amount of nanoparticle reinforcements as can be seen from Fig. 6.

### Antimicrobial performance

#### Antialgal growth analysis

**Figure 7:**
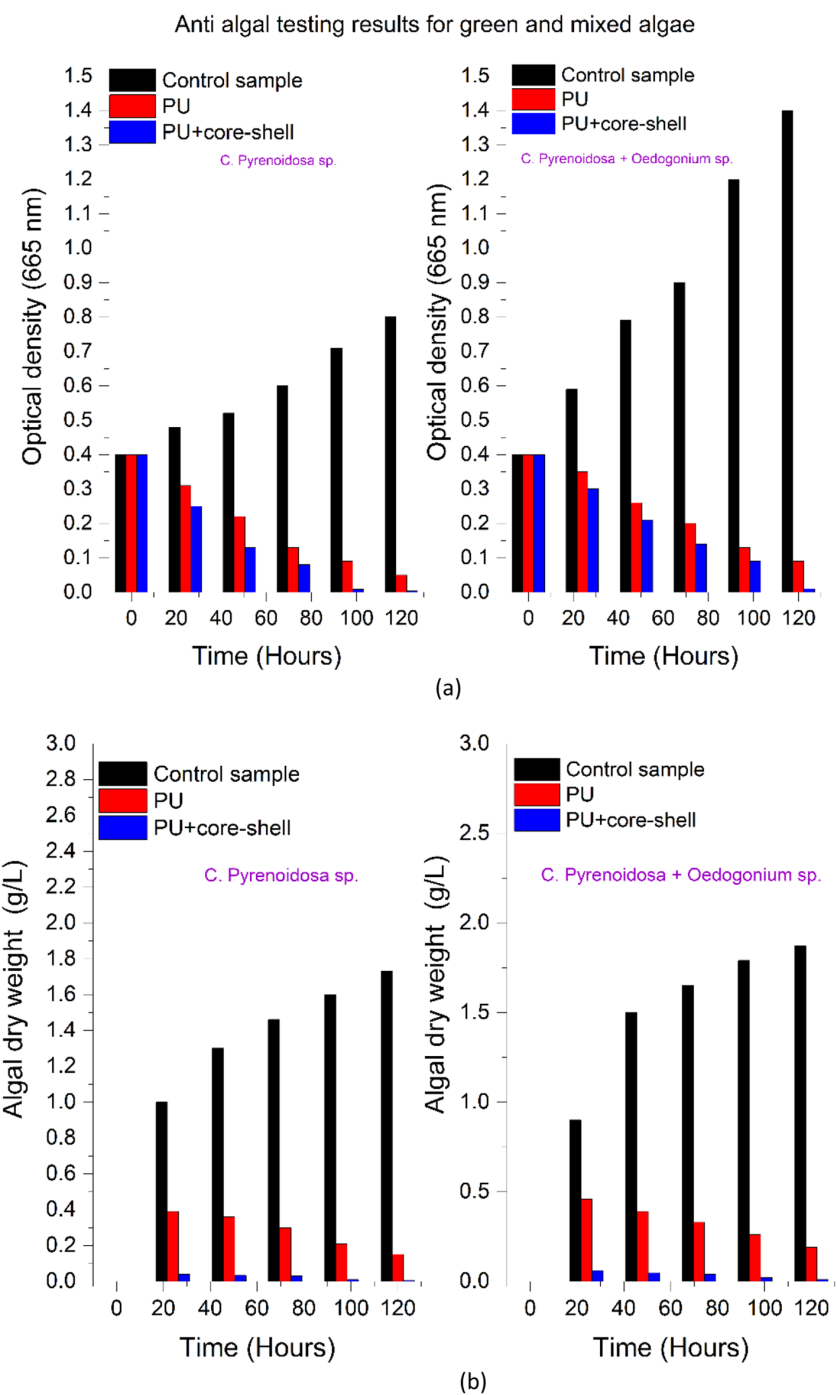

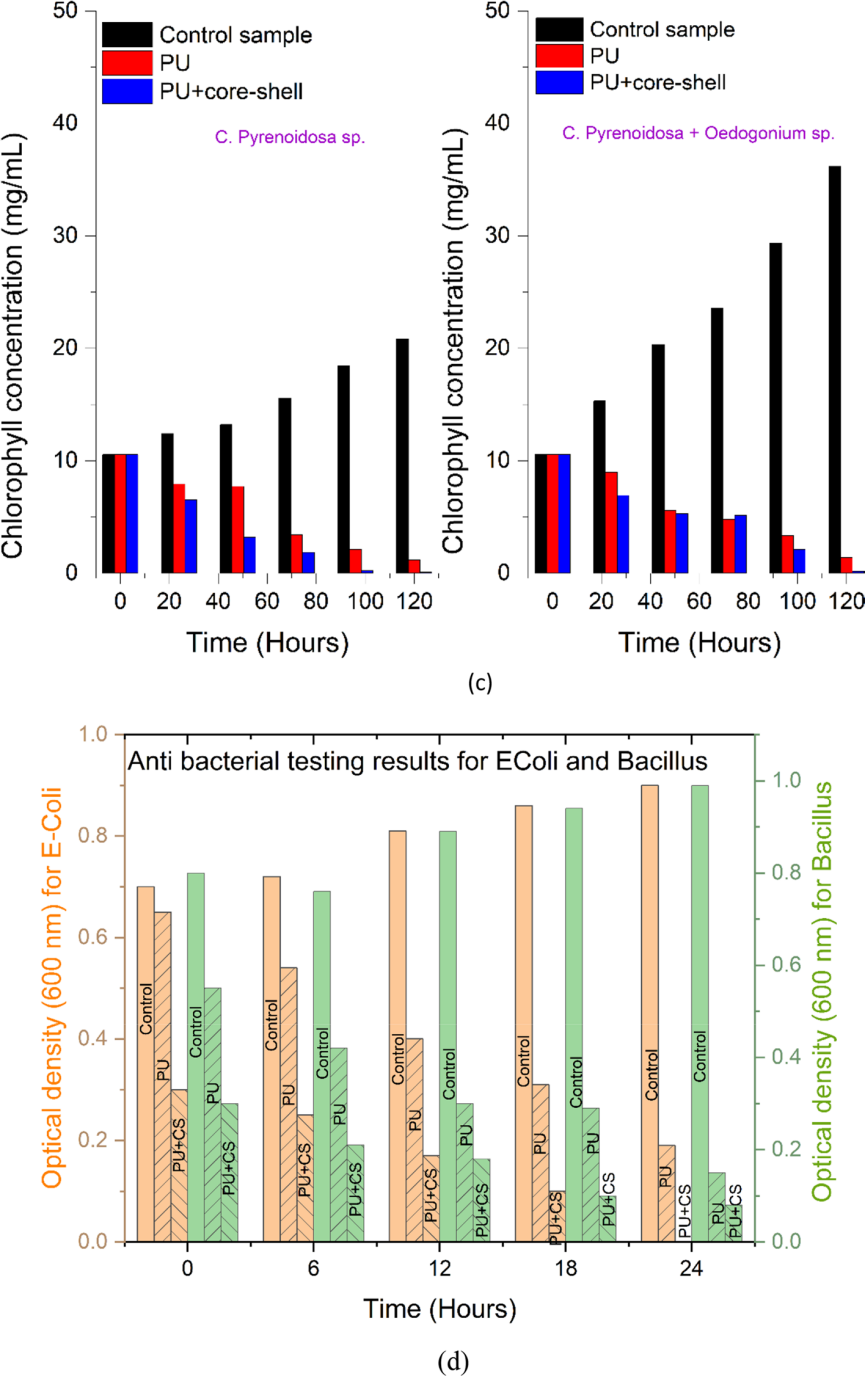

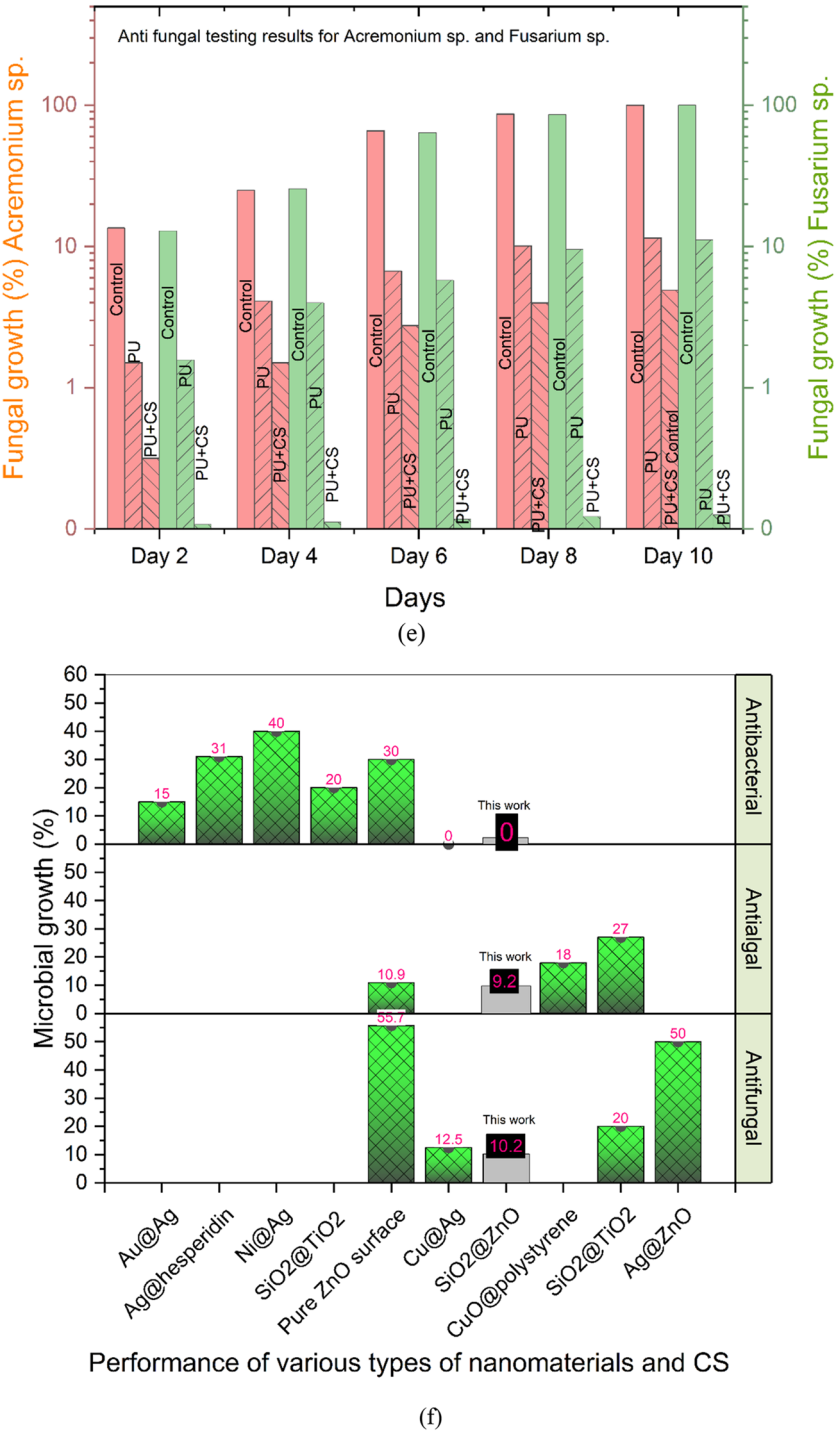
Anti-algal effect against green algae (*Chlorella pyrenoidosa*) and mixed algae (*C. pyrenoidosa* + *Oedogonium* sp.) shown through (**a**) optical density. (**b**) algal dry weight. (**c**) chlorophyll concentration. (**d**) Antibacterial study of coatings against *E. coli* and *Bacillus* through measurement of optical density in the culture media. (**e**) Antifungal study of coatings against *Acremonium* and *Fusarium* species. (**f**) Benchmarking of the results obtained in this work with use of SiO_2_@ZnO CS incorporated PU coating against various coatings reported in literature providing antifungal, antibacterial and antialgal performances. The comparison here also includes pure ZnO surface which showed significant differences against SiO_2_@ZnO core shell surface. Au@Ag^30^, Ag@Hesperiding^31^, Ni@Ag^32^,SiO_2_@TiO_2_^11,14^, Pure ZnO^33^, Cu@Ag^34^, CuO@polystyrene^35^, Ag@ZnO^36^. Image processed using Origin Software (https://www.originlab.com/).

The green algae (*C. pyrenoidosa*) and mixed algae (*C. pyrenoidosa* and *Oedogonium* species) were grown for 120 h and their growth was monitored every 24 h (Fig. 7a). Also, the algal dry weight and chlorophyll concentration were measured. The tests included monitoring of two types of algal strains on three samples (control sample, PU sample and PU + CS sample).

Algal dry weight was measured by centrifugation of the sample. Obtained pallets were suspended in methanol and shaken. Further, the sample was placed in the water bath at 60 °C for 15 min before being centrifuged again. At this point, the biomass of the algae was weighed (see Fig. 7b). As chlorophyll is present in all green plants including algae, it is important to calculate the amount of total chlorophyll present in the coated sample to validate the antialgal study. Chlorophyll A and B play an important role in absorbing light for photosynthesis. Chlorophyll A's central role is as an electron donor in the electron transport chain. Chlorophyll B's role is to give organisms the ability to absorb higher frequency blue light for use in photosynthesis. This research estimated chlorophyll A and chlorophyll B through the standard formulae^29^, the results of which are shown in Fig. 7c:

1 $$Chlorophyll \,A \,({\mathrm{mg}}/{\mathrm{ml}})=16.72\times {OD}_{665.2}-9.16\times {OD}_{652.4}$$

2 $$Chlorophyll \, B\, ({\mathrm{mg}}/{\mathrm{ml}})=34.092\times {OD}_{652.4}-15.28\times {OD}_{665.2}$$

3 $$Total \, chlorophyll \, concentration \,({\mathrm{mg}}/{\mathrm{ml}}) \,= \, Chlorophyll \,A \,({\mathrm{mg}}/{\mathrm{ml}})+ \,Chlorophyll \, B \, ({\mathrm{mg}}/{\mathrm{ml}})$$

#### Bacterial growth analysis

Optical density was measured at 600 nm (the standard OD rate for *E. coli* and *Bacillus*) every 6-h for up to 24 h. The polyurethane-based coating created with SiO_2_@ZnO core–shell nanoparticles at 4% (wt) concentration of nanoparticles showed the most resilient anti-bacterial performance. The SiO_2_@ZnO incorporated PU coating demonstrated a 100% reduction against *E. coli* and a 90% reduction against *Bacillus* (see Fig. 7d).

#### Fungal growth analysis

Testing of antifungal properties involved two species namely, *Acremonium* and *Fusarium.* The fungal analysis was performed for upto 10 days*.* The results for the two species on the control sample, PU sample and PU + CS are shown in Fig. 7e. The presence of SiO_2_@ZnO was observed to inhibit the growth of fungus. On the assumption that the growth diameter and weight measurement of the fungus in the control Petriplate was 100%, the growth reduction of the fungus in the Petriplate containing the nanocoatings was estimated. At 4 (wt%) concentration, the SiO_2_@ZnO core–shell based PU coatings demonstrated 95% fungal growth reduction against *Acremonium* sp. and 99.9% against *Fusarium* sp*.*

After analyzing the antibacterial, antifungal and antialgal results on SiO_2_@ZnO individually, we prepared a comparative chart shown in Fig. 7f to compare the antimicrobial performance of various types of core–shell nanoparticles as well as pure ZnO reported in previously published literature (Au@Ag, Ag@Hesperidin, Ni@Ag, SiO_2_@TiO_2_, Pure ZnO, Cu@Ag, CuO@polystyrene, Ag@ZnO) core–shell nanoparticles. It can be inferred from Fig. 7f that Ag@Hesperidin, Ni@Ag and SiO_2_@TiO_2_ core–shell nanoparticles show microbial growth of upto 31%, 40%, 20% against *E. coli* bacteria whereas the CS of SiO_2_@ZnO developed in this work completely inhibit (0% growth) the bacterial growth. Similarly, various other antimicrobial studies were analyzed in terms of fungus and algae with different types of nanomaterials. By far, the SiO_2_@ZnO incorporated coating developed in this work outbids all the other combinations in terms of antimicrobial performance.

### Mechanical characterisation

#### Taber testing

One of the traditional techniques used to assess the abrasion resistance of a surface is the Taber test. Taber test measures the abrasion triggered by the plastic deformation as per relevant standards ASTM D1044 and D4060. The Taber test requires a flat sample of about 100 mm^2^ (square or round). The sample is placed on a turntable platform which is rotated at a fixed speed around a vertical axis. To accomplish this testing, Taber 5135 Abraser (Supplementary Fig. S3) was utilised during this investigation. During the tests, the abrasive wheels are rotated while applying a normal load and lowered onto the specimen surface. By rubbing the test specimen against the sliding revolution of the two abrasive wheels, distinctive rubbing wear can be noticed. During the test, a vacuum system collects the wear debris because of abrasion during the process. The Taber experiments were done using CS10 calibrase wheels which are appropriate to test organic coatings for a load of upto 500 g. The mass loss counted after every 500 cycles can be inferred as the loss of material due to abrasion and this can suitably be used to index the wear resistance of the two surfaces developed in this work. An area of about 30 cm^2^ was covered by the ensuing abrasion marks, which take the appearance of a circular band with crossed arc patterns. A load of 500 g and about 2500 cycles were used in this work to compare PU and PU + CS (SiO_2_@ZnO) coatings at 4 wt% concentration.

### Quantification of abrasion resistance

**Figure 8:**
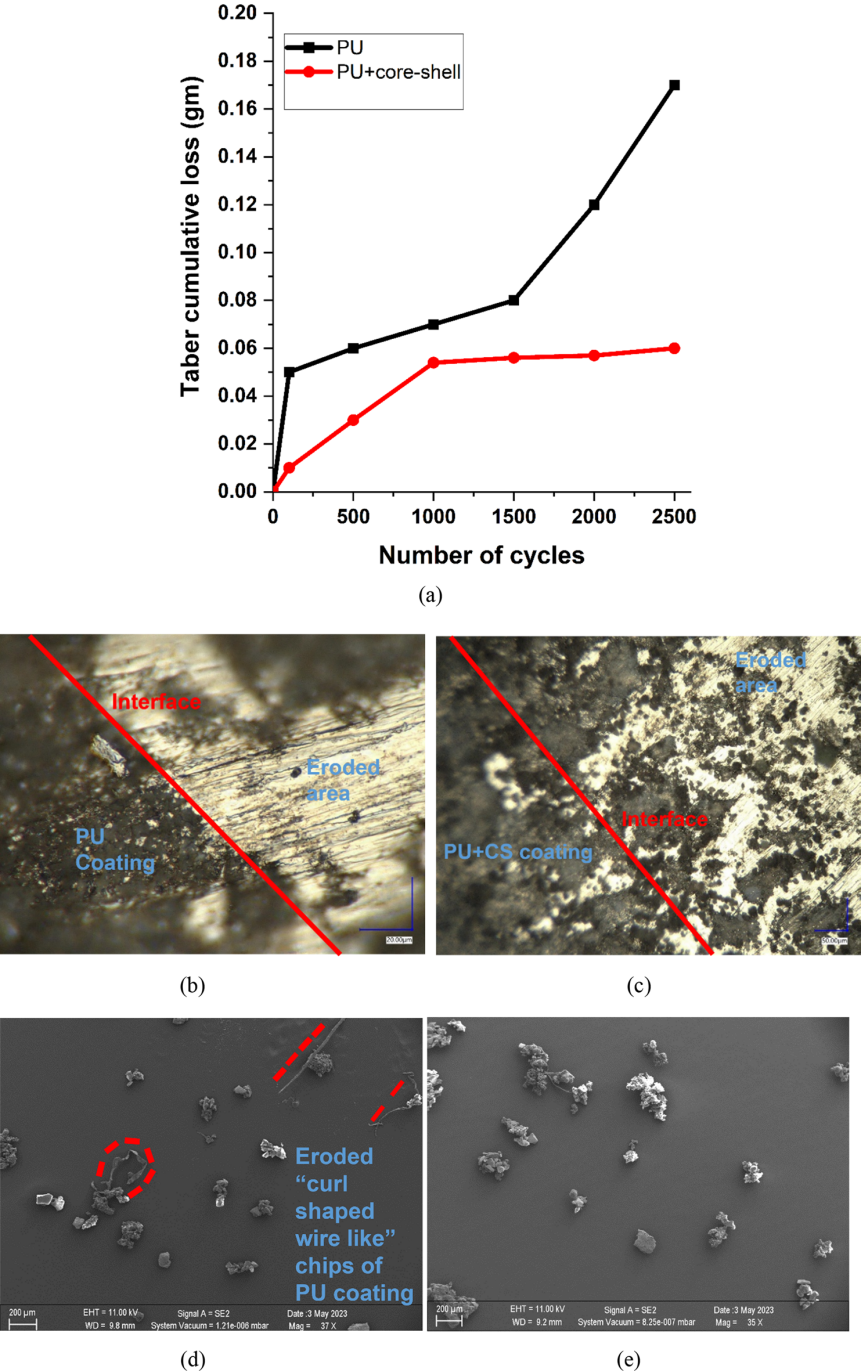

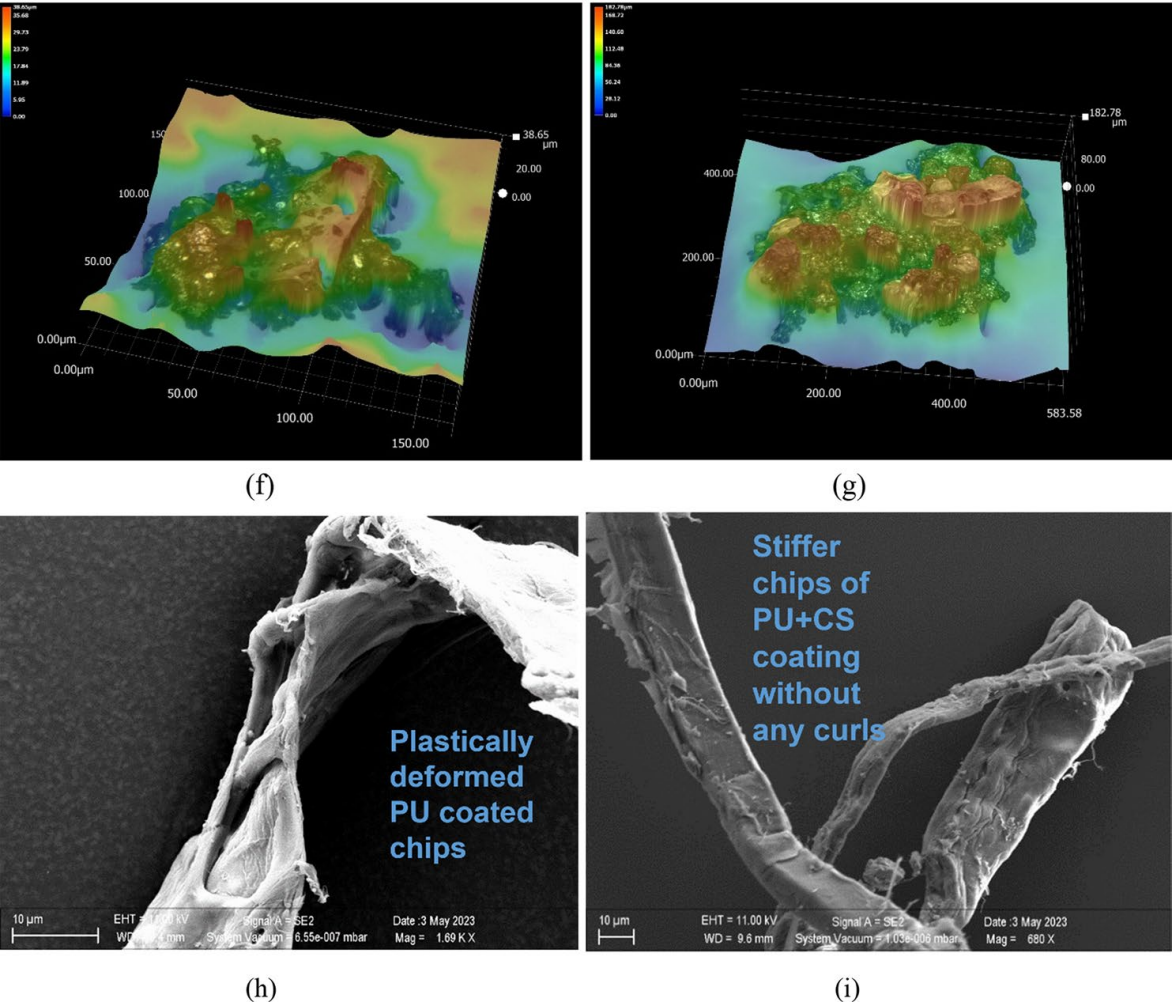
(**a**) Cumulative wear loss measured through Taber test on two coating types: PU and PU + CS. (**b–e**) Light microscopy and SEM images revealing the modes of material removal during Taber Abrasion in the PU surfaces and (**f–i**) in the CS (SiO_2_@ZnO) incorporated PU surface.

The capacity of a coated surface to withstand the abrasion load caused by erosion or impact is known as abrasion resistance. The sample of coatings subjected to 5135 rotary platform abraser revealed the wear performance of both surfaces as indicated in Fig. 8a. Figure 8a shows the cumulative wear loss for both surfaces after completing 2500 cycles at a load of 500 g. The analysis of data performed for upto 2500 cycles showed that the presence of core–shell nanospheres in the PU media improved its abrasion resistance in comparison to the pristine PU coating. It was expected considering that the SiO_2_ core has significantly better mechanical strength than the PU polymer. From a physical inspection of the abraded samples, some remnants of distinct modes of material removal mechanism were observed in the two types of surfaces (PU and PU + CS). It was therefore considered relevant to inspect the wear debris of the two test surfaces through optical microscopy and through scanning electron microscopy imaging. These results are shown in Fig. 8b–i by comparing the abraded debris of pure PU vs CS incorporated PU.

Inspection of the PU surface eroded by Taber as shown in Fig. 8b using light microscopy compared to the eroded Taber surface of PU + CS coating shown in Fig. 8c highlights that the eroded metal surface can clearly be seen in the former case but not as clear in the latter case. This speaks for the fact that the material removal mechanism in the former case was swift and that the removal was somewhat frustrated in the latter case when PU was mixed with core–shell nanospheres. The SEM image comparisons of the wear-debris in both cases Fig. 8d, e show the unique presence of wire like ribbons and curl chips of PU which indicated significant plastic deformation during abrasion. However, the abraded chips of the PU + CS material were seen to be visibly stiffer which not only resisted curling of the chipping but also prevented thinning of chips during deformation, which was a contrasting feature of the PU chips shown in Fig. 8f–i. This highlights the fact that the PU was less strongly bonded to the base material and had weaker adhesion under the shear loading conditions since Taber abrasion creates a state of shear stress, whereas the incorporation of CS nanospheres in PU significantly improved the shear resistance of the CS incorporated coating making it difficult for the Taber wheel to abrade the coating cleanly from the surface. Thus, it highlights the ability of core shell SiO_2_@ZnO to improve the adhesion with the steel surface, which is a positive outcome. The main reason for an improved adhesion is a chemical modification of the nanoparticle surface which is carried out by functionalisation of core–shell nanoparticles using a coupling agent (APTES) which is also known as an adhesion promoter. These coupling agents are mainly bi-functional reactive additives, designed in such a way that one part of the molecules forms covalent bonds with the substrate and another part participates in the crosslinking of the binder system of the paint during film formation to create strong substrate-coating adhesion.

### Scanning acoustic microscopy (SAM) of the coated test samples

Since the early 1980s, SAM has been used as an NDT test method for inspecting manufacturing flaws and delamination to provide imaging of the internal structure of various materials^37,38^. The transducer emits a short ultrasonic pulse and then detects the echo. If the part is free of defects, there will be two signals, from the near and far surface. If there is an internal defect, such as a void or delamination, the transducer will detect an additional return signal^39^.

This research made use of a scanning Acoustic Microscope (SAM) developed by PVA TePla, Germany for the inspection of the X-section of the coated substrates. SAM comes equipped with an H2 PreAmplifier. To perform the SAM, the coated samples were submerged in a deionised water tank and an ultrasonic transducer was moved over the sample to collect the data. A PT75-3–12.7 transducer having a diameter of 3 mm with a focal distance of 12.7 mm at a peak frequency of 75 MHz was used for the measurements. SAM works by directing focused sound from a transducer at a small point on a target object. Sound hitting the object is either scattered, absorbed, reflected (scattered at 180°), or transmitted (scattered at 0°). It is possible to detect the scattered pulses travelling in a particular direction.

**Figure 9:**
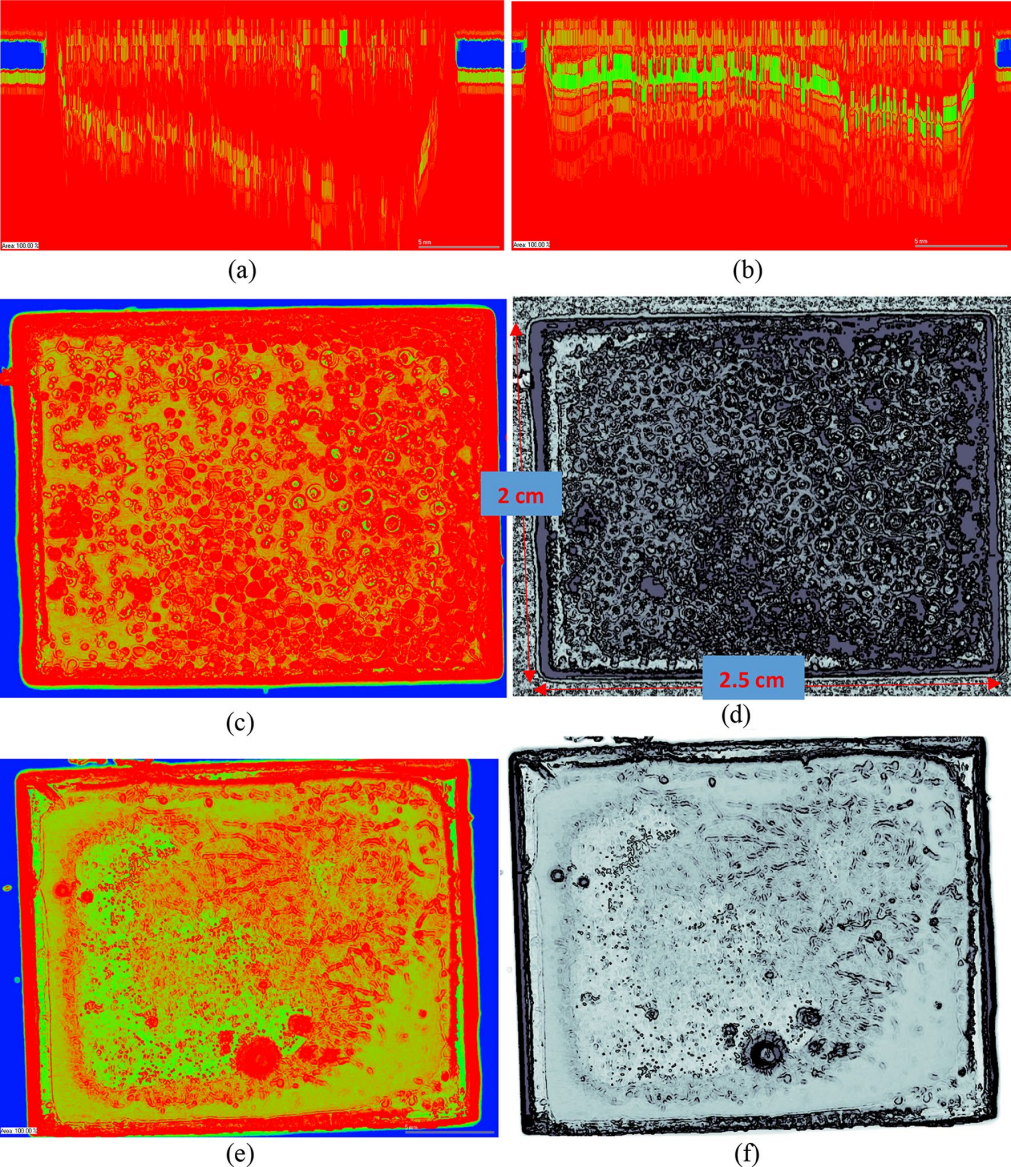
SAM image of the coated surfaces in B-scan and C-scan modes. (**a**) B-scan (vertical X-section) of PU sample. (**b**) B-scan (vertical X-section) of PU + CS sample. (**c**,**d**) C-Scan of the PU sample imaged by the SAM on top of the coated surface. (**e**,**f**) C-Scan of the PU + CS sample imaged by the SAM on top of the coated surface.

It is a sophisticated technology that requires specialised knowledge and experience to operate and understand the data, but it still has some advantages over other NDT technologies that are currently available, which makes it a great choice in particular situations. Defect inspection to identify voids, and microcracks, aroused the need for the implementation of SAM imaging as part of this work^40^. Accordingly, the study used a machine tool SAM 300 PVA TePla^41^ to explore its utility in the detection of delamination, non-homogeneous layers and air gaps in the developed coating samples. Figure 9a, b highlight a comparison of the two types of coated surfaces in the B-scan (vertical scan mode) under the SAM transducer. Figure 9a is an image of a PU coated sample while Fig. 9b is a PU + CS (SiO_2_ + ZnO) sample. What becomes immediately clear by the comparison is that the presence of core–shell nanospheres in the coating makes the sample more receptive to the transducer signals and provides a much stronger signal response. The blue colour in Fig. 9a, b depicts the water surface level—the depth at which the samples were submerged in the water. Thus, the length between the two water columns denotes the width of the coated sample which can be seen to contain a variety of flaws and defects. To collate further information about these coated samples, C-scan (lateral scans) were made into both samples the outcome of which is shown in Fig. 9c–f.

C-scan inspection from SAM for the PU-coated sample shown in Fig. 9c, d revealed ring-shaped defect structures pointing to the microstructural morphology of the PU coating. Contrary to this, the CS-incorporated PU shown in Fig. 9e, f showed chains shaped features. The internal inspection of these coated structures points to the wide array of differences in the material's microstructure and the fact that the abrasion resistance of PU was lower than the PU + CS samples coated surfaces would point to the fact that the ring-shapes defects in the PU sample tend to make the sample weaker. Contrary to this, the chain-shaped feature seen in the PU + CS configuration of the coating indicates the strength, possibly alluding to improved cross-linking as those chains indicate improved polymeric bonding—however, this aspect would need further examination. This preliminary investigation proves that the CS-incorporated PU can achieve better properties through changes in the microstructure and these aspects were well summarized throughout the paper.

## Conclusions

We report the design, synthesis and testing protocol of a novel polymeric coating materials system utilising the concept of core–shell nanospheres which upon mixing with the PU polymer media provided the best antimicrobial performance reported in the literature to date, whilst achieving a good adhesion and strong abrasion resistance. Tested through Taber abrasion tests (ASTM D1044 and D4060), the presence of the core–shell structures of SiO_2_@ZnO developed in this work showed a marked improvement in the hydrophobicity when tested against a water droplet test and improved antialgal, antibacterial and antifungal behaviour. The scanning acoustic microscopic examination of the two coating systems revealed for the first-time ring and chain-shaped features that can qualitatively describe the strengthening mechanisms in polymers. Also, the light microscopy and SEM inspection of the Taber wear debris indicated frustrated wear mechanisms in the SiO_2_@ZnO incorporated PU coating as opposed to pure PU coating, thus highlighting the mechanical strengthening of the coated surface. Due to these benefits, we find the developed coating system immensely useful for protecting steel surfaces in marine transportation. Our results revealed that the reported coating system outbids the previously reported SiO_2_@TiO_2_ core–shell based coating system.

## Supplementary Information

Supplementary Figures.

## Data Availability

The datasets used and/or analysed during the current study are available from the corresponding author on reasonable request.
