# Supplementary material for: Fabrication and testing of a multifunctional SiO2@ZnO core–shell nanospheres incorporated polymer coating for sustainable marine transport
Source: Sci Rep. 2023 Jul 29;13:12321. doi: 10.1038/s41598-023-39423-9 (PMC10387051; doi:10.1038/s41598-023-39423-9)
Supplement: Supplementary file 1 — Supplementary Figures. [file 41598_2023_39423_MOESM1_ESM.docx]

**Supplementary information:**


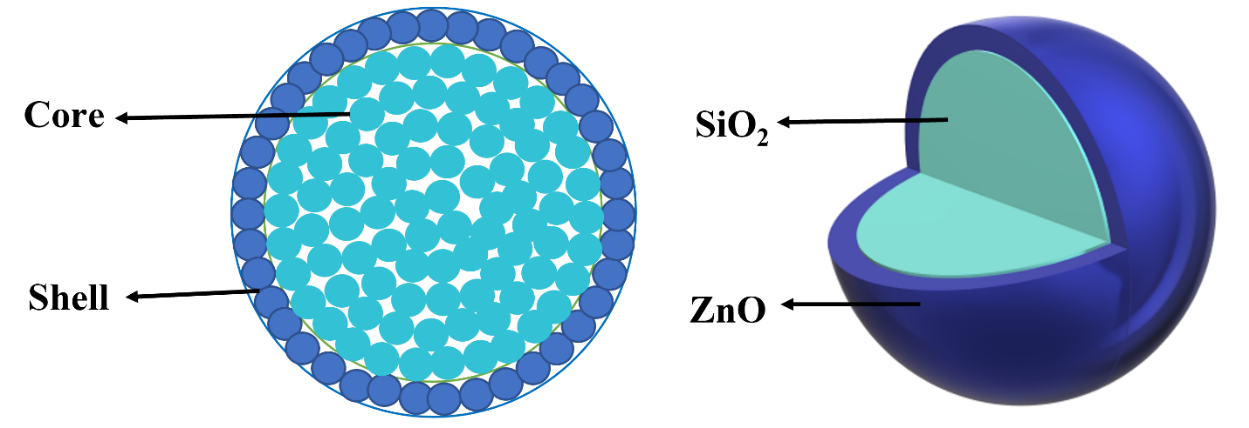


**Fig.S1:** Schematic of core-shell structure


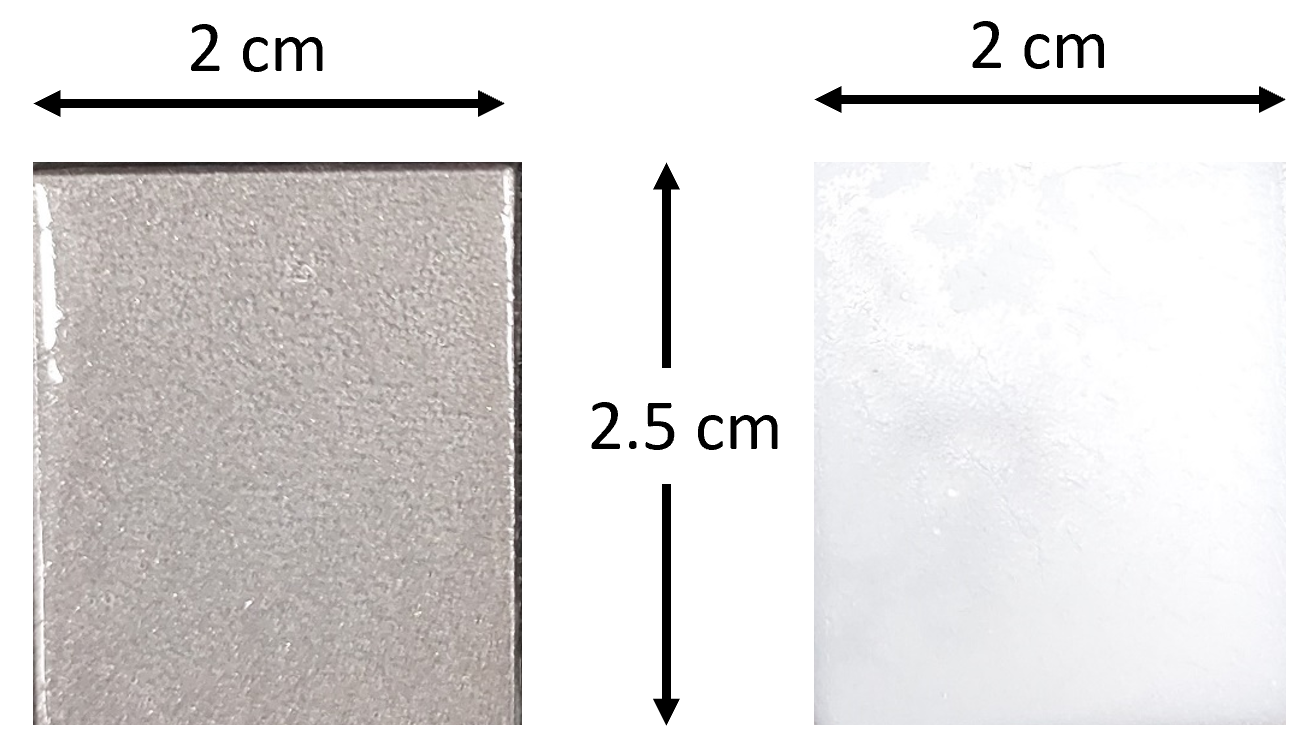


1. (ii)

(d)

**Fig.S2:** Coated steel surfaces (2.5 x 2 cm^2^) (i) PU coating and (ii) Silica@ZnO coreshell nanospheres incorporated PU coating


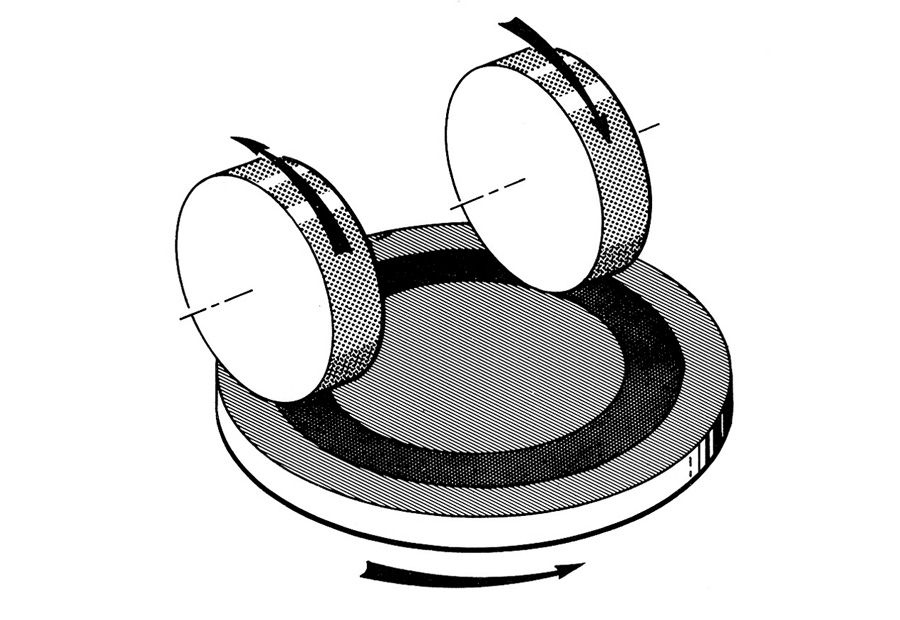


Test sample

CS 10 Calibrase wheels

CS 10 Calibrase wheels

(a)


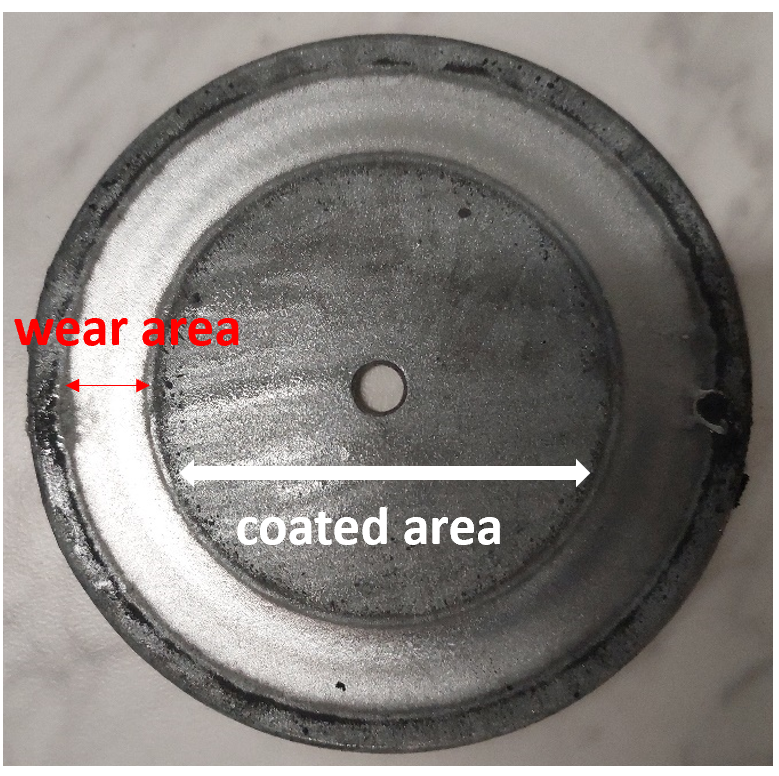

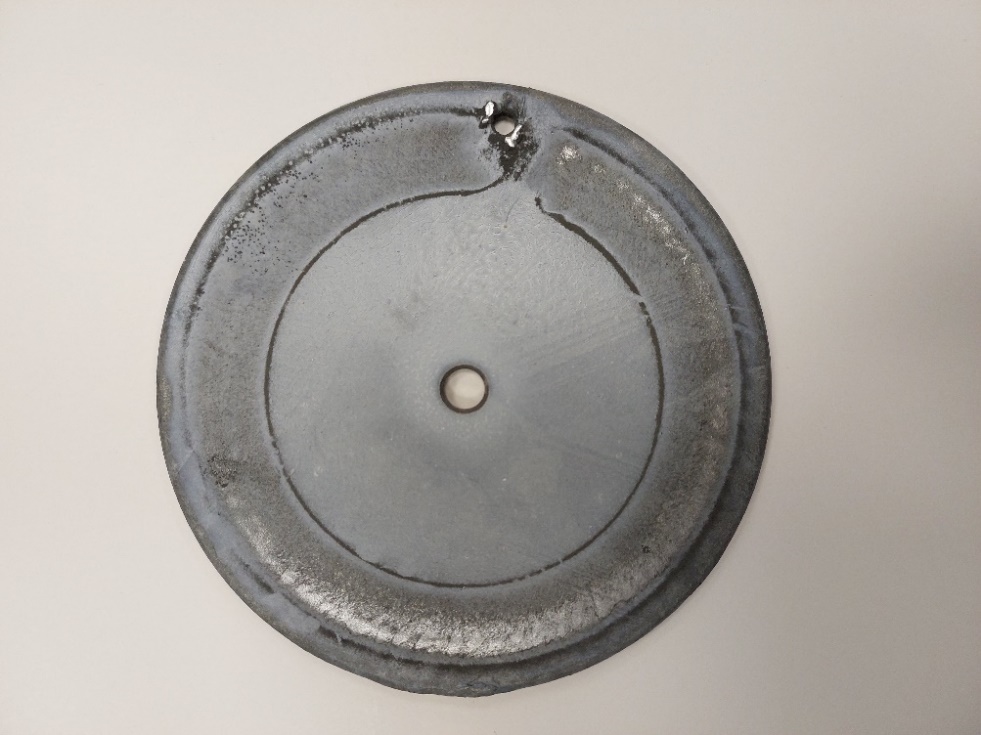


(i) PU Coating (ii) PU + CS Coating

(b)

**Fig. S3.** (a) Taber 5135 abraser testing principle (b) Test samples for Taber tests (i) Pure polyurethane (PU) coating (ii) SiO_2_@ZnO nanosphere incorporated PU coating
